# Autochthonous faecal viral transfer (FVT) impacts the murine microbiome after antibiotic perturbation

**DOI:** 10.1186/s12915-020-00906-0

**Published:** 2020-11-20

**Authors:** Lorraine A. Draper, Feargal J. Ryan, Marion Dalmasso, Pat G. Casey, Angela McCann, Vimalkumar Velayudhan, R. Paul Ross, Colin Hill

**Affiliations:** 1 APC Microbiome Ireland, University College Cork, Cork, Ireland; 2 School of Microbiology, University College Cork, Cork, Ireland; 3 Present Address: SAHMRI, North Terrace Adelaide, 5000 South Australia; 4 Present Address: Normandie Univ, UNICAEN, ABTE, 14000 Caen, France

**Keywords:** Bacteriophage, Virome, Transfer, Microbiome, Antibiotic, Murine, Bacteriome

## Abstract

**Background:**

It has become increasingly accepted that establishing and maintaining a complex and diverse gut microbiota is fundamental to human health. There are growing efforts to identify means of modulating and influencing the microbiota, especially in individuals who have experienced a disruption in their native microbiota. Faecal microbiota transplantation (FMT) is one method that restores diversity to the microbiota of an individual by introducing microbes from a healthy donor. FMT introduces the total microbial load into the recipient, including the bacteria, archaea, yeasts, protists and viruses. In this study, we investigated whether an autochthonous faecal viral transfer (FVT), in the form of a sterile faecal filtrate, could impact the recovery of a bacteriome disrupted by antibiotic treatment.

**Results:**

Following antibiotic disruption of the bacteriome, test mice received an FVT harvested prior to antibiotic treatment, while control mice received a heat- and nuclease-treated FVT. In both groups of mice, the perturbed microbiome reverted over time to one more similar to the pre-treatment one. However, the bacteriomes of mice that received an FVT, in which bacteriophages predominate, separated from those of the control mice as determined by principal co-ordinate analysis (PCoA). Moreover, analysis of the differentially abundant taxa indicated a closer resemblance to the pre-treatment bacteriome in the test mice that had received an FVT. Similarly, metagenomic sequencing of the virome confirmed that faecal bacteriophages of FVT and control mice differed over time in both abundance and diversity, with the phages constituting the FVT persisting in mice that received them.

**Conclusions:**

An autochthonous virome transfer reshaped the bacteriomes of mice post-antibiotic treatment such that they more closely resembled the pre-antibiotic microbiota profile compared to mice that received non-viable phages. Thus, FVT may have a role in addressing antibiotic-associated microbiota alterations and potentially prevent the establishment of post-antibiotic infection. Given that bacteriophages are biologically inert in the absence of their host bacteria, they could form a safe and effective alternative to whole microbiota transplants that could be delivered during/following perturbation of the gut flora.

## Background

The mammalian gastrointestinal tract is home to a complex and intimately associated microbial ecosystem (microbiota) comprised of bacterial, archaeal, fungal, protist and viral components. The virome is mainly composed of bacteriophages (bacterial viruses collectively referred to as the phageome). It has been estimated that the human gut can contain as least as many bacteriophages as there are bacteria (10^14^) [1–3], making it perhaps the most densely populated ecological niche in nature. Co-evolution over millennia has selected those members of the microbiota that either cause no harm or confer a benefit to the host; these are commonly referred to as the commensals and mutualists of the gut [4–6]. The gut harbours abundant and diverse bacterial species [7], and bacteriophages may play an important role by restricting overgrowth of the most successful strains. This ecological model is termed ‘kill the winner’, whereby lytic bacteriophages prey preferentially on their most abundant host and proliferate. Following this, another bacterial species may emerge as the most prevalent for a time, until its population is likewise diminished by the phage [8]. If phages have the potential to modulate the gut bacteriome, then in turn they could have an impact on host–microbe interactions and on host health [9]. Mammalian hosts have evolved to rely on microbial activities to assist digestion, provide vitamins, resist pathogens, and regulate metabolism and the immune system [10, 11]. The intestinal microbiota is also a potent source of antigens and potentially harmful compounds, including carcinogens [12]. Obtaining an appropriate microbiota at birth and subsequently developing and maintaining it in the face of challenges such as antibiotic therapy is an important determinant of health and wellbeing [13].

Antibiotics can be just one example of how essential medical therapies can lead to perturbations in the gut ecosystem. Broad spectrum antibiotics have saved millions of lives but may leave the gut microbiota in an altered state in which the delicately constructed ecosystem of the gastrointestinal tract may be compromised.

Probiotics [14–16], prebiotics and other dietary interventions have been investigated [17] as tools to ameliorate the negative aspects of antibiotic use. The most radical therapy is faecal microbiota transplantation (FMT), whereby the microbiota of an individual is supplemented by that of a healthy donor [18]. In human therapy, FMTs are usually confined through practitioner guidelines to those suffering from severe, moderate or recurring *Clostridium difficile* infections [19].

With a standard FMT, the entire stool is homogenised and filtered through gauze and the resulting preparation is then infused into the gut. The entire microbiota is introduced into the recipient, including bacterial, archaeal, fungal and viral species, albeit some species do not survive processing due to their oxygen sensitivity. Several studies have looked at the transfer and engraftment of donor bacteriophages into the microbiota of recipients following FMT [20, 21]. Even more interesting however are a number of recent studies have suggested that faecal bacteriophages alone have the ability to modulate the gut microbiota and its function [22, 23]. Therefore, we investigated if autochthonous viral transfer (in which bacteriophage predominate, following removal of all other cellular microorganisms) can modulate the bacteriomes and phageomes of mice following disruption with antibiotic treatment.

## Results

### Sequencing of the murine bacteriome

Two separate trials (designated as Study 1 and Study 2) were performed sequentially, with some variation between the two studies, so that the effect of bacteriophages on the microbiome could be determined as a reproducible intervention. In both trials, 16S rRNA amplicon sequencing was used to determine the composition of the faecal bacteriome of pre- and post-antibiotic treated mice (*n* = 16). Half of the mice subsequently received an FVT (*n* = 8) or a heat- and nuclease-treated FVT (hnFVT) as a control (*n* = 8). Viral transfer was conducted using a bacteria free-virus faecal filtrate collected from the same cohort prior to antibiotic treatment. In Study 1, a single gavage was performed after antibiotic washout, while in Study 2 a second gavage was also performed 4 days later (see experimental design in Additional file 1: Fig. S1). MiSeq (V3 kit - 300 bp paired-end reads) sequencing resulted in an average of 16,805 ± 8079 (Study 1) or 31,758 ± 64,605 (Study 2) paired-end reads across the samples, and an average of 14,514 ± 7095 (Study 1) or 29,350 ± 59,214 (Study 2) individual reads post-quality filtering (Additional file 2: Tables S1 and S2).

### Impact of antibiotics on the murine gut bacteriome

**Fig. 1:**
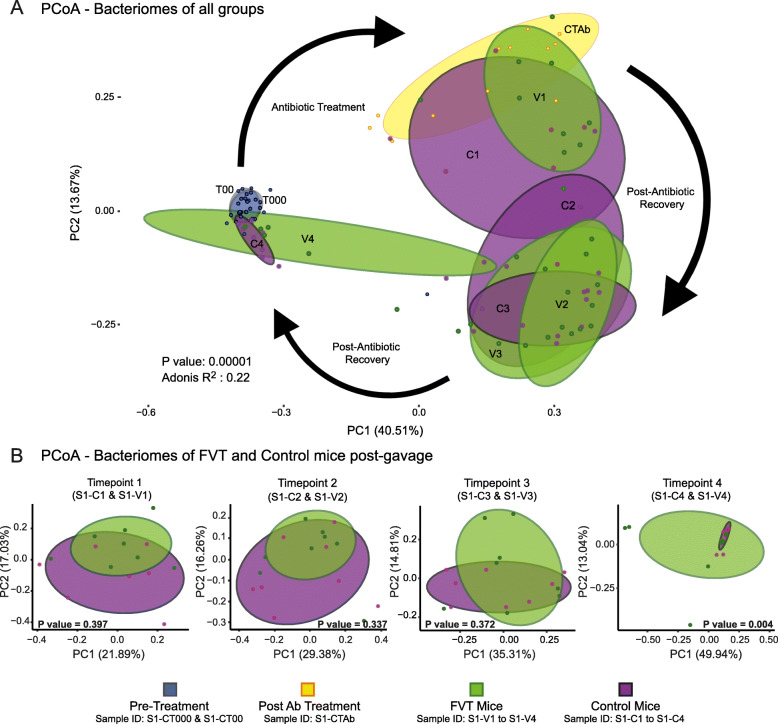
PCoA plots of unweighted UniFrac distances for all timepoints and groups in Study 1. Ellipses represent 70% confidence intervals (**a**). Subsequently, a UniFrac PERMANOVA test was performed with the Adonis function to determine the statistical differences between FVT and Control mice (model formula = antibiotic treatment timepoint + phage/control status), the relevant PCoA plots and *P* values are displayed (**b**)

After a period of acclimatisation, mice were administered an antibiotic cocktail of penicillin and streptomycin in their drinking water for either 4 days (Study 1) or 2 days (Study 2). Treatment time in Study 2 was reduced by half following visualisation of the dramatic impact of the antibiotic in Study 1. Following antibiotic treatment (designated as S1-CTAb in Study 1 and S2-CTAb in Study 2), a significant change occurred in the murine bacteriome as compared to pre-treatment mice (samples S1-CT000/S2-CT000 and S1-CT00/S2-CT00). These differences can be visualised via unweighted UniFrac PCoA (principal co-ordinate analysis; Fig. 1a), as well as via weighted UniFrac and Bray–Curtis PCoA (Additional file 3: Fig. S2).

Using the Shannon index as a measure of alpha diversity, it is apparent that the bacterial diversity decreased after antibiotic treatment in both studies. In Study 1, after 4 days of antibiotic treatment (visualised as sample S1-CTAb) (Additional file 4: Fig. S3A), there is a lower Shannon diversity than that observed for S2-CTAb in Study 2 (Additional file 4: Fig. S3B), which is consistent with a shorter 2-day administration of antibiotics. However, when we examine bacterial cell numbers (log 16S copies/gram of faeces; Additional file 5: Fig. S4), there was a greater reduction in cell numbers in Study 2, when antibiotics were only administered for half the time (Study 1: log (16S copies/gram of faeces) mean 9.45, median 9.93, and Study 2: mean 7.80, median 7.49). It must be noted however that in Study 2 the alpha diversity decreased further than that observed for the S2-CTAb sample in the Control and FVT Mice in samples taken post-FVT/hnFVT gavage (samples S2-C1 and S2-V1), and concomitantly, bacterial numbers were elevated (Control mice (S2-C1): mean 8.56, median 8.76, and FVT Mice (S2-V1): mean 9.08, median 9.25) as was observed for Study 1-CTAb, where the lowest Shannon diversity of this study was also observed.

The increase in bacterial cell numbers may correlate with the outgrowth of certain species (Additional file 6: Fig. S5). For example, in Study 1, we observed an increase in *Escherichia coli*/*Shigella* species in the antibiotic-treated mice that continued to increase in the post-gavage mice (S1-C1/S1-V1). After the shorter exposure to the antibiotic treatment in Study 2, this out-growth of *E. coli*/*Shigella* species was only observed in the faeces of the 10-h post-gavage mice (S2-C1/S2-V1). In the S2-CTAb mice, we did however observe an increase in “Unclassified” species (those operational taxonomic units (OTUs) unclassified to genus level) and a decrease in *Alistipes*, *Bacteroides*, *Barnesiella* unclassified Bacteroidetes and unclassified Firmicutes species. Classification of OTUs to a phylum level ascribes these changes to a decrease in Bacteroidetes and Firmicutes, and an increase in Proteobacteria, Cyanobacteria and unclassified others. However, it is to be noted that inter-sample variation was observed during cell number quantification. This could be due to errors incurred during sample weighing, due to cell density differences or simply could reflect variation across animals within the study; for this reason, median compositional values were used to examine the bacterial profiles of animals in the various cohorts.

### The impact of bacteriophage on the antibiotic-treated murine gut bacteriome

#### Study 1

Following antibiotic treatment, mice were divided into two groups (*n* = 8) and received a gavage of either autochthonous viable phages (FVT group) or heat- and nuclease-treated bacteriophages (Control group) by means of a sterile faecal filtrate. In Study 1, the inferred PCoA plots of unweighted Unifrac distances (Fig. 1a) show separation of the bacteriomes of Control and FVT mice into different clusters that both gradually return to a location similar to that of the pre-antibiotic bacteriome. Eleven days post-gavage, there is a significant difference in the faecal bacteriomes of FVT and Control groups as revealed by Adonis PERMANOVA analysis (*P* value = 0.004) (Fig. 1b). These differences were also observed using Bray–Curtis distances but not using weighted Unifrac, which may imply that some of the significant differences are due to low abundant taxa given the contrast between weighted and unweighted Unifrac matrices (Additional file 3: Fig. S2A, Additional file 2: Table S3).

**Fig. 2:**
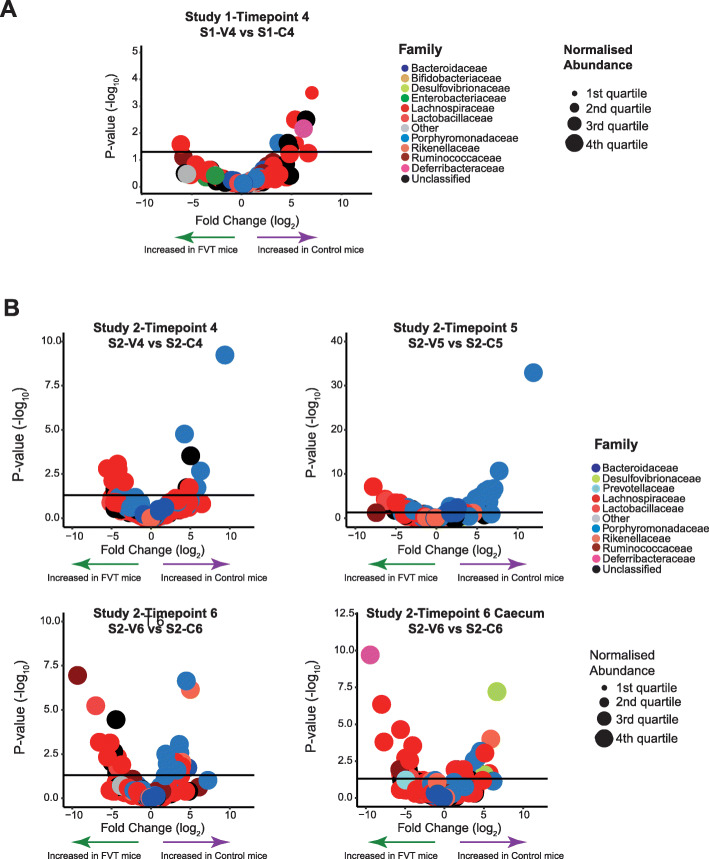
Volcano plots showing the results of DESeq2 which detects differentially abundant OTUs between the FVT and Control mice at each timepoint in Study 1 (**a**) and Study 2 (**b**). OTUs with an adjusted *P* value < 0.05 are positioned above the horizontal line. Normalised abundance is also represented. Families belonging to the phylum Firmicutes are indicated via varying shades of red, while families derived from Bacteroides are visualised in blue, and additional colours are used to represent other families and phyla

Differentially abundant taxa were identified between the FVT (sample S1-V4) and Control (sample S1-C4) groups 11 days post-gavage (Fig. 2a). The abundance of these twelve OTUs was statistically distinct between the two cohorts and appeared to be low level in terms of the community relative abundance (Additional file 7: Fig. S6 and Additional file 2: Table S4). Of these 12, six belong to the family Lachnospiraceae, while Ruminococcaceae accounted for one, as did Porphyromonadaceae and Deferribacteraceae. The final three were unclassified to the family level but are of the Order Clostridiales. Interestingly, all 12 OTUs not only differed in their abundance between S1-C4 vs S1-V4 mice, but all are statistically different in their abundance compared to the pre-treatment mice (S1-CT00). With 10 of these OTUs increased in Control mice (that did not receive viable phage), it implies that the FVT aided in restoring the murine bacteriome to more closely resemble its pre-treatment state. The OTUs increased in Control mice were predominantly of the family Lachnospiraceae; the remainder included the species *Mucispirillum schaedleri* and *Parabacteroides goldsteinii*. Only two OTUs were increased in mice receiving phage and these belong to the families Lachnospiraceae and Ruminococcaceae and include the species *Eubacterium siraeum*.

#### Study 2

Study 1 demonstrated that administration of a bacteriophage enriched gavage can impact the murine microbiota and so we performed a second more comprehensive study. Here a second viable/heat-treated FVT gavage was administered 4 days after the first (at day 12 of the trial) and an increased number of samples (including caecum content) were analysed.

**Fig. 3:**
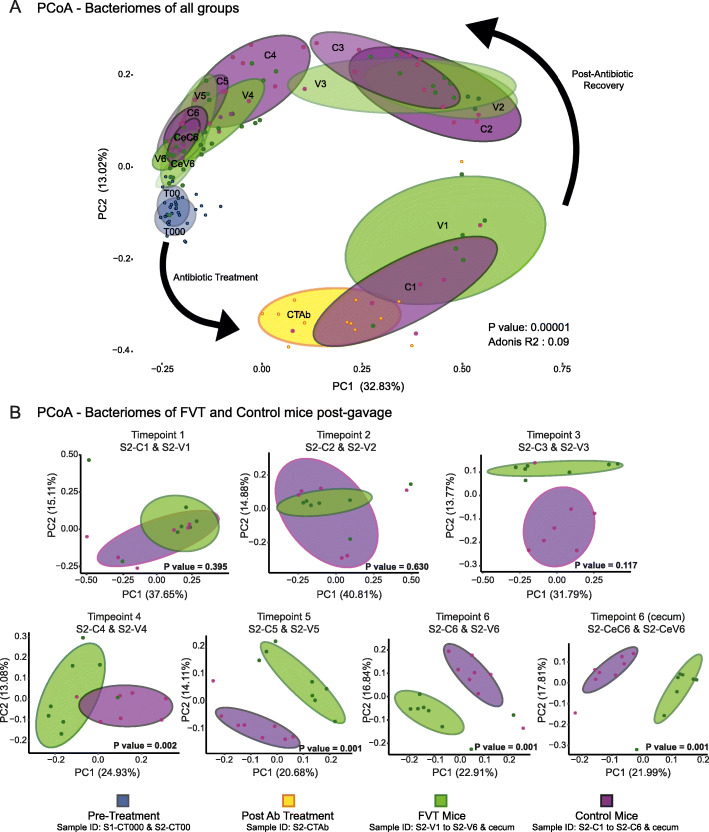
PCoA plots of unweighted UniFrac distances for all timepoints and groups in Study 2. Ellipses represent 70% confidence intervals (**a**). Subsequently, a UniFrac PERMANOVA test was performed with the Adonis function to determine the statistical differences between FVT and Control mice (model formula = antibiotic treatment timepoint + phage/control status), the relavant PCoA plots and *P* values are displayed (**b**)

In Study 2, PCoA plots (unweighted Unifrac, Bray–Curtis and weighted Unifrac (Fig. 3a and Additional file 3: S2B)) display clear clustering of OTUs defined by whether they received heat killed or viable phage. As in Study 1, over time the bacteriome of the mice gravitates from the post-antibiotic treatment state (S2-CTAb) to the pre-treatment state (S2-CT00 & S2-CT000). We observed separation of the microbiota of mice into significantly different clusters from 4 days post-second gavage (timepoint 4) to the end of the experiment (timepoint 6, 15 days post initial gavage), where these differences were also revealed in the caecum (S2-CeC6 and S2-CeV6) (Adonis PERMANOVA: timepoint 4 *P* value = 0.002, timepoint 5 *P* value = 0.001, timepoint 6 *P* value = 0.001, timepoint 6 (caecum) *P* value = 0.001) (Fig. 3b). These differences were also observed using Bray–Curtis distances but not in the case of weighted Unifrac, which again may imply that some of the significant differences are contributed by less abundant taxa given the contrast between weighted and unweighted Unifrac matrices (Additional file 3: Fig. S2B, Additional file 2: Table S3).

Alpha diversity confirms a clear increase in diversity post-antibiotic treatment in both control and FVT-receiving mice (Additional file 4: Fig. S3B). Several differentially abundant taxa were observed between S2-C4 and S2-V4 (13 OTUs), S2-C5 and S2-V5 (51 OTUs), S2-C6 and S2-V6 (37 OTUs) and S2-CeC6 and S2-CeV6 (25 OTUs) (Additional file 7: Fig. S6 and Additional file 2: Table S5). Twenty-six OTUs were noted to be differentially abundant across two or more timepoints and five were highlighted to be differentially abundant at timepoint 6 in both faeces and caeca, revealing that these core taxa are more directly influenced by the presence or absence of administered bacteriophages. With respect to these 31 OTUs, the taxa were almost equally distributed between Bacteroidetes (52%) and Firmicutes (48%). At the family level, Porphyromonadaceae (36%) and Lachnospiraceae (32%) predominated. Lactobacillaceae accounted for a further 7% of OTUs, while Rikenellaceae and Ruminococcaceae accounted for 6% and 3%, respectively. The most commonly assigned genus was *Barnesiella* at 16%; however, 65% of OTUs could not be assigned to the genus level. Of the *Barnesiella* identified, 75% were classified to be of the species *Barnesiella intestinihominis*. It appears that the administration of an FVT led to subtle but significant changes in the microbiota, with less abundant OTUs being most affected.

**Fig. 4:**
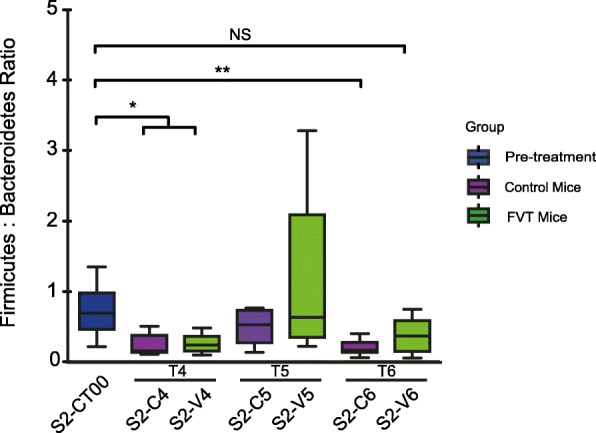
Bacteroidetes Firmicutes ratio reveals that the ratio is statistically higher in FVT mice as determined via Kruskal–Wallis with Dunn’s multiple comparison post-test; CT00 vs CT4/PT4: *P* value < 0.05; CT00 vs CT6: *P* value < 0.01; CT00 vs PT6: *P* value > 0.05)

A volcano plot was used to visualise the differential association of OTUs across the timepoints and in the caecum (Fig. 2b). Firmicutes family members (red hues) are more abundant in FVT animals across the final two timepoints and in the caecum, while Bacteroidetes family members (blue hues) are more abundant in Control mice. If we compare the faecal Firmicutes–Bacteroidetes ratio with pre-treatment (S2-CT00) mice, we observe a significant decrease in this ratio in both sub-sets at timepoint 4 (8 days post initial gavage). This ratio decreases further in Control mice at timepoint 6 (15 days post initial gavage) while those that received bacteriophage return to that observed for pre-treatment mice at timepoint 6 (Kruskal–Wallis with Dunn’s multiple comparison post-test; S2-CT00 vs S2-C4/S2-V4: *P* value < 0.05; S2-CT00 vs S2-C6: *P* value < 0.01; S2-CT00 vs S2-V6: *P* value > 0.05) (Fig. 4). Such statistical changes were not observed in preliminary Study 1.

Further examination revealed that certain taxa remain increased in FVT or Control mice over time (Additional file 7: Fig. S6 and Additional file 2: Table S5). For example, OTU 8 belonging to the family Porphyromonadaceae fluctuates in abundance across the three faecal timepoints represented here but is consistently increased in Control mice. All differentially abundant OTUs as determined by DESeq2 were compared to the abundance of these same OTUs in pre-treatment mice (S2-CT00). Of the differentially abundant taxa increased in FVT mice at the end of the experiment (timepoint 6), only 14% of faecal OTUs and 36% of caecally derived OTUs differed in their abundance as compared to pre-treatment animals, while 87% and 50% respectively of differentially abundant OTUs in Control mice differed from pre-treatment mice. (Additional file 2: Table S5—OTUs that differ from S2-CT00 mice are highlighted in blue; Additional file 7: Fig. S6—the names of OTUs that differ in their abundance from S2-CT00 levels are written in blue font for FVT mice and red text for Control mice).

### Murine viromes

In addition to examining the impact of bacteriophages on the murine bacteriome, we also performed a metagenomic study on DNA isolated from viruses extracted from the murine faecal samples. Current methodology for virome extraction requires more faecal matter than can be obtained from a single murine faecal pellet; therefore, the results represent the viromes of groups of mice rather than individual animals, in that faecal samples were pooled for pre-treatment (*n* = 16), antibiotic-treated (*n* = 16), control (*n* = 8) and FVT-treated mice (*n* = 8) at each timepoint (Additional file 1: Fig. S1). Despite our best efforts however in Study 2, it was still necessary to incorporate a whole genome amplification step which may introduce a bias toward amplification of small circular viruses [24]. The virome data was analysed utilising an assembly-based approach as described in other studies we have published [20, 25, 26].

**Fig. 5:**
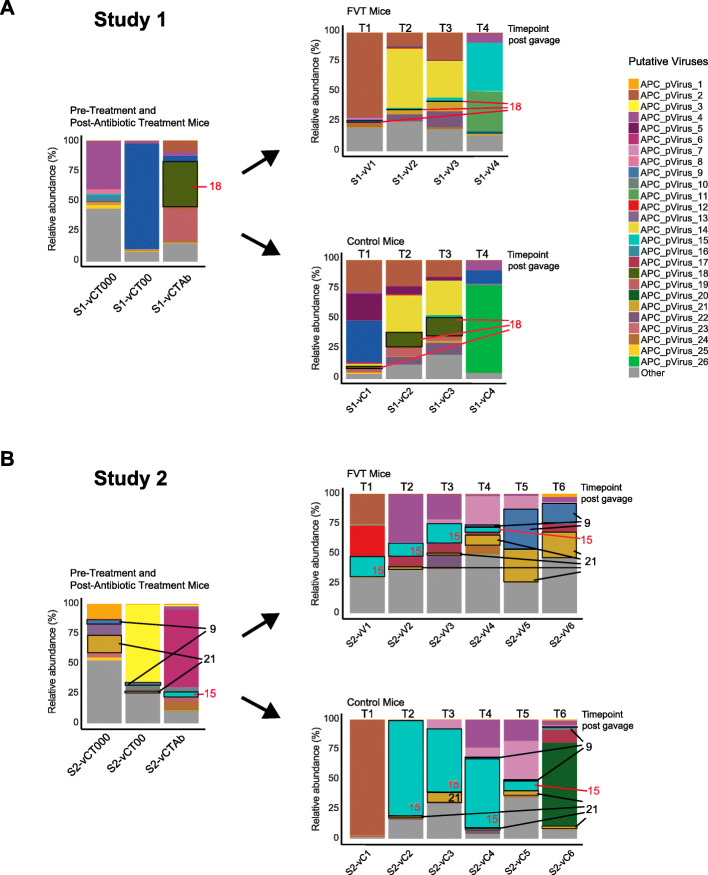
Metagenomic analysis on DNA isolated from viruses extracted from murine faecal samples in Study 1 (**a**) and Study 2 (**b**). Results reflect the viromes of groups of mice, where faecal samples were combined and represent the viral pool in pre-treatment (*n* = 16), antibiotic-treated (*n* = 16), Control (*n* = 8) and FVT mice (*n* = 8) at each timepoint. Following sequencing contigs were assembled into putative viruses and the relative abundances are represented within this bar plot. Certain contigs of interest have been highlighted; those in black text are found in abundance in pre-antibiotic treatment mice and in greater abundance in FVT mice than in Control mice. Those highlighted in red text are in greater abundance in post-antibiotic treated mice and in greater abundance in Control mice than FVT mice

The virome that made up the FVT gavage is visualised as that present in the S1-CT000/S2-CT000 and S1-CT00/S2-CT00 samples (Fig. 5). In both studies, the virome composition appears to be different between Control and FVT mice in the post-gavage samples. Moreover, in study 2, from 8 days post-gavage treatment, those mice that received a viable FVT retain a notable abundance of the bacteriophages introduced in the gavage. For example, APC-pVirus9 and APC-pVirus21 are found in abundance in FVT mice from timepoint 4 where they are all but diminished in the control. The opposite trend is observed in both studies when it comes to tracking viral contigs seen in high abundance in the post-antibiotic treatment samples (S1-CTAb and S2-CTAb); these contigs appear to be retained in higher abundance in Control animals that did not receive an influx of viral particles rather than in those that received the FVT. For example, in Study 1, APC-pVirus18 is seen in high abundance following antibiotic treatment and is retained at higher abundance in Control animals up to timepoint 3. Similarly, in Study 2, APC-pVirus15 is seen to be in much higher abundance in Control animals from timepoints 2–5 than in FVT mice.

Within the individual studies, we have reported that at specific timepoints differentially abundant bacterial taxa were detected. In Study 1, we observed that in the final timepoint, (where such differentially abundant bacterial taxa were observed) the viromes of control and FVT mice differ not only in content but also in their corresponding relative abundances. A total of 152 bacteriophages were identified in the eight Control mice at this timepoint (T4), while 163 are associated with FVT-receiving mice (Fig. 5). With respect to viral abundances at this final timepoint, APC-pVirus11 and APC-pVirus15 constitute the majority of the viral load of FVT mice, while APC-pVirus26 predominates in the gut of Control mice. In Study 2, where two sequential gavages of viable FVT were administered, the variability in the profiles of the putative bacteriophages present and their relative abundances between Control and FVT animals appears to differ even more. The richness of viral diversity is also elevated in FVT mice, especially in the final timepoints in tandem with the observation of differentially abundant bacterial taxa. This equates to a 20–30% increase in specific bacteriophages in these animals up to 11 days post gavage (see Additional file 2: Table S6). It must be noted however that these results are based on a pooled sample, and whole genome amplification was performed in Study 2, thus no statistical conclusions can be drawn.

## Discussion

The impact of externally added phages on GI tract bacteria has been studied for over 100 years Since then, there have been multiple studies revealing the impact of selected phage on a range of GI pathogens [2, 9, 27–31]. Recently, Hsu et al. [32] demonstrated in a murine model the profound impact of bacteriophages on the composition and function of a simplified gut microbiota colonised in germ-free mice. Phages were seen to influence strains beyond that of their hosts, modulating the metabolome and reshaping the overall community structure.

Here we report on how the administration of an autochthonous faecal virome transplant (FVT) had a significant impact on the bacteriome of mice following antibiotic-induced disruption, in two separate mouse experiments. The bacteriome of FVT-receiving animals differed in the abundance and/or diversity of species and FVT mice appeared to retain the bacteriophage they received up to 11 days (Study 1) or 15 days (Study 2) post gavage, long after the transit time of several hours noted for bacteriophage through the GI tract [33]. Following the administration of a single FVT gavage in Study 1, we witnessed an alteration in the faecal bacteriome. This reshaping of the gut bacteriome was also observed to a greater extent in Study 2 where the bacteriophage dose was doubled by means of sequential gavages 4 days apart. Differentially abundant taxa were observed across the final three faecal timepoints and subsequently in the caeca after sacrifice, with the majority of taxa found to be in similar abundance in FVT mice to that observed in pre-antibiotic treated mice. Samples derived from the murine caecum have a bacteriome composition distinct from that of the faecal samples [34]. Therefore, differences observed here between FVT-treated and Control mice indicate alterations in the bacteriome at multiple GI sites and suggest a widespread impact of bacteriophages on the murine gut. When the relative abundance of the differentially abundant OTUs was examined, it revealed that individually they contributed to only a low percentage of the overall community. However, when these OTU reads are examined at a higher taxonomic rank, they collectively resulted in a statistical change in the Firmicutes–Bacteroidetes ratios of the FVT mice restoring the ratios to the pre-antibiotic treatment state. This indicates that autochthonous bacteriophages have impacted and shaped the bacteriome, restoring it to a composition more closely resembling the pre-treatment state. By killing bacteria, bacteriophages implement population fluctuations in their bacterial hosts; they significantly influence global biochemical cycles and are also considered to be crucial in driving microbial species diversity due to the fact that they are species-specific.

When we look at the abundance differences at a species level however, it is interesting that *Barnesiella intestinihominis* appeared on multiple occasions to be present in greater abundance in Control mice (Study 2—T4, T5, T6 and caecum T6). This Gram-negative commensal bacterium has been classified as an oncomicrobiotic for its immunomodulatory properties [35]. Loss of this species in FVT-receiving mice suggests that they obtained a bacteriophage that could utilise *B. intestinihominis* as a host, thus diminishing the population of this species. This is described as the “kill the winner” model, but what also may be the case is the “kill the relative” model, whereby the induction or presence of prophage/lysogenic bacteriophages can trigger an epidemic among susceptible bacterial competitors [36]. Several other models also exist that describe how bacteriophage can shift the dynamics of bacterial populations. One model of interest and relevance to this study is the “community shuffling model”, which describes the induction of prophage by environmental factors such as sub-inhibitory levels of antibiotics, such as quinolones or beta-lactams (as was utilised here) from bacterial species such as *E. coli* [37], *C. difficile* [38], *E. faecalis* [39] and *Staphylococcus aureus* [40]. Prophage induction will alter the gut environment and microbiota profile via lysis of bacterial species and as a consequence of differing host immune system interactions [36]. Should the presence of antibiotics within the current studies have encouraged such events, it should have occurred prior to administration of exogenous bacteriophages and so would have been present in both control and test animals. These prophages could however have contributed to the bacteriophage profile present in S1-CTAb/S2-CTAb mice and to the initial ecology to which the administered FVT was introduced.

The benefits of faecal transplantation as a medical intervention have been well documented; the impact of bacteriophage within this microbial community has yet to be fully investigated. The transfer and long-term colonisation of bacteriophages species during standard FMT has been reported [20], with temperate phage members of *Siphoviridae* found to be transferred between human donors and recipients with significantly greater efficiency than other bacteriophage groups [41]. One report found that a sterile filtered faecal transplant (FFT), where cellular microbes were removed, produced longitudinal changes in the bacterial and viral community structures of faecal samples in five human recipients suffering from *C. difficile* infections, all of whom recovered following treatment [23]. The potential role of bacteriophage in the recovery of the microbiota from the ravaging effects of this infection was acknowledged, along with potential roles for bacterial components and metabolites. Furthermore, a study by Rasmussen et al. details that an allochthonous FVT from lean donors modulated the microbiome, decreasing symptoms of type 2 diabetes and obesity in a murine model [22]. This establishes bacteriophage as effective modulators of the microbiota beyond that of recovery from post-antibiotic infection and may indicate they play a role in efficacy of FMT.

Each gram of human faeces contains approximately 10^11^ bacterial cells, ~ 10^11^ bacteriophages, ~ 10^7^ colonocytes, ~ 10^8^ archaea, ~ 10^8^ viruses, ~ 10^6^ fungi, protists and metabolites [36, 42, 43]. The protocols used to process the murine faeces and prepare the FVT gavage in this study would have removed all biological species apart from bacteriophages and viruses (and those metabolites or microbial structures present in the control gavage). Heat induced capsid disassembly/denaturation of bacteriophages and the subsequent use of nucleases to destroy viral nucleic acids [44, 45] ensures that the Control mice would have only received small-molecule metabolites, bacterial components or antimicrobial compounds of bacterial origin (e.g. bacteriocins) that contribute to the normal intestinal microenvironment. Thus, we can deduce that the alterations to microbiome (both bacteriome and virome) of test mice are due to the acquisition of viable bacteriophages.

While our observations agree with the published literature [23], we should note the limitations associated with this work. Importantly, our use of a single autochthonous combined faecal sample for each group is a limiting factor in estimating biological variability. Furthermore, while our microbiota profiling techniques are widely used in the literature, they are based on the 16S rRNA gene which is limited in taxonomic resolution. Similarly, the qPCR quantification approach is prone to errors and variability and so should not be considered a reflection of absolute abundance. However, even with these in mind, we believe these studies are key initial experiments in investigating the potential of FVT as a medical intervention.

## Conclusions

Here we used an autochthonous FVT to demonstrate that bacteriophages can re-model the murine gut microbiota following antibiotic treatment. This supports another well-controlled study by Rasmussen et al., 2019, which indicates that an allochthonous FVT (from lean donors) can modulate the microbiota, decreasing symptoms of type 2 diabetes and obesity in a murine model [22]. Further to this, Ott et al. [23] have suggested the role of bacteriophages in human FFT (faecal filtrate transplant [23]) in disease resolution in rCDI patients. Such reports in addition to our current study validate a role for the transplanted phageomes in microbiota population dynamics in the gut. Currently FMT largely focusses on transferring living microbes to those in gastrointestinal distress. This study suggests that an FVT may have a role in prophylaxis, addressing post-antibiotic treatment disruption of the microbiota, re-balancing the microbial balance and such could potentially prevent the establishment of post-antibiotic infection. Given that bacteriophages are inert biological entities incapable of colonising in the absence of a sensitive host, they could form a viable alternative to whole microbiota transplants, which could be delivered as a robust formulation during/following perturbation of the gut flora.

## Methods

### Mouse models and experimental design

#### Study 1

Sixteen BALB/c mice were obtained from Harlan Laboratories UK Ltd. and were housed within the Biological Services Unit, University College Cork (UCC). Mice were received at 7–8 weeks of age and allowed to acclimatise for 5 days on a standard rodent diet. During this time, faecal samples were collected, frozen (− 80 °C) and used, in part, to prepare FVT phage-rich material which will be used for oral gavage. This required obtaining 2–3 g of faecal pellets which were resuspended in a solution of 10-ml filter sterilised SM buffer (50 mM Tris-HCl; 100 mM NaCl; 8.5 mM MgSO_4_; pH 7.5) and 2 ml 1 M NaHCO_3_ (to help deacidify the stomach). After vortexing, the solution is centrifuged at 4700 rpm for 20 min. The supernatant was then filter sterilised (0.45-μM filter) and divided into 2 separate containers: one to be administered to the test (FVT) group and contains active bacteriophages/viruses, while the second was heated at 95 °C for 15 min, followed by DNase (Ambion) treatment at 37 °C for 1 h (according to the manufacturer’s guidelines) as described by Reyes et al. [46], and thus contained inactivated bacteriophages to be administered to the control group. After acclimatisation, all 16 mice were administered antibiotic treatment (penicillin 1000 U/ml and streptomycin 3 g/L, in order to disrupt both Gram-positive and Gram-negative species) in their drinking water for 4 days (and faecal samples were collected) followed by 1 day of antibiotic wash-out, where standard drinking water was administrated ad libitum. Following this, the mice were divided into 2 groups and housed with other members of their group (*n* = 8), faecal samples were collected and the FVT group were gavaged with 0.2 ml of the FVT material (as prepared above) while the control group received inactivated bacteriophages. Faecal samples were then collected 10 h, 24 h, 34 h and 11 days afterward.

#### Study 2

Study 2 was performed as that described for Study 1 with the following deviations. Sixteen BALB/c mice were received at 8–10 weeks of age and allowed to acclimatise for 3 days. After acclimatisation, all 16 mice were administered antibiotic treatment (penicillin 1000 U/ml and streptomycin 3 g/L) in their drinking water for 2 days followed by 1 day of antibiotic wash-out. Following this, the mice were divided into 2 groups (*n* = 8), faecal samples were collected and the FVT group were gavaged with 0.2 ml of active bacteriophages (as prepared in Study 1, albeit with faecal material originating from Study 2 mice) while the control group received inactivated bacteriophages (“Gavage 1”). Faecal samples were then collected 1 and 4 days afterward. Forthwith a second gavage (“Gavage 2”) was subsequently administered 4 days after the first (at day 12 of the trial) in a similar manner as described for Gavage 1. Again, faecal samples were collected from both groups post-FVT gavage at 1, 4, 7 and 14 days. Post-mortem, caecum content was also collected.

### DNA extractions and library preparation for MiSeq

Faecal samples were all frozen immediately after collection at times indicated in Additional file 1: Fig. S1 and then used to extract bacterial DNA for 16S rRNA analysis of the bacteriome (~ 2–5 faecal pellets were collected from each mouse at each timepoint and used to track alterations in the bacteriome of each individual mouse). The QIAamp DNA Stool Mini Kit (Qiagen, Hilden, Germany) was used according to manufacturer’s guidelines to extract bacterial DNA but was modified to include a bead-beating step. 16S ribosomal DNA hypervariable regions V3 and V4 were amplified via PCR using a high fidelity polymerase (Phusion; Thermo Fisher Scientific) and the primers V3F (341F) - 5′-CCTACGGGNGGCWGCAG-3′ and V4R (805R) – 5′-GACTACHVGGGTATCTAATCC-3′ [47] with the addition of the appropriate Illumina Nextera XT overhang adapter sequences (Illumina, San Diego, CA, USA). Following purification using a magnetic bead capture kit (Ampure; Agencourt), the amplicon libraries underwent a second PCR reaction to attach dual indices and Illumina sequencing adapters using the Nextera XT index kit (Illumina, San Diego, CA, USA). Following purification (as described above), the dsDNA libraries were quantified using a Qubit® Fluorometer (Thermo Fisher Scientific) and were then pooled in equimolar concentrations. Ready to load libraries were sequenced on an Illumina MiSeq (Illumina, San Diego, California) using V3 sequencing kit (300 bp paired-end reads) at GATC Biotech AG, Germany.

### Analysis of 16S rRNA gene amplicon sequencing data

The quality of the raw reads was visualised with FastQC v0.11.3. Forward and reverse reads were merged using FLASH [48]. Primers were removed from the merged reads using the fastx_truncate command of USEARCH [49]. The trimmed reads were then demultiplexed with a Phred quality score of 25 using the split_libraries_fastq.py script of the QIIME package [50]. The demultiplexed sequences in FASTA format were then de-replicated using the derep_fulllength command of USEARCH and sequences below a minimum length cutoff (400 nt) were removed. Singletons were removed using the minsize option of the sortbysize command of USEARCH. The resulting sequences were then clustered into OTUs using the cluster_otus command and then chimera filtered using both the de novo and reference-based chimera filtering implemented in USEARCH with the ChimeraSlayer gold database v20110519 [49]. The reads were then aligned back to the OTUs using the usearch_global command of USEARCH to generate a count table which was input into R for statistical analyses. Taxonomy was assigned to the sequences using mothur v1.38 [51] against the RDP database version 11.4, as well as classified with SPINGO to species level [52]. Only OTUs with a domain classification of Bacteria or Archaea were kept for further analysis. A phylogenetic tree of the OTU sequences rooted on the midpoint was generated with FastTree [53]. Alpha diversity and Beta diversity were generated using PhyloSeq v1.16.2, which also was used for a principal co-ordinate analysis as implemented in Ape v3.5. Differential abundance analysis was carried out with DESeq2 v1.12.4 [54]. All visualisation in R was performed with ggplot2 v2.2.1. Permutational multivariate analysis of variance (PERMANOVA) was performed in R Vegan package with the adonis function (Model formula = antibiotic treatment timepoint + Phage/control status) with 9999 permutations.

### Real-time qPCR

Quantification of bacterial load was performed to determine by assessing the number of 16S rRNA genes present per gram of faeces using LightCycler® 480 apparatus (Roche), associated with LightCycler® 480 Software (version 1.5; Roche), was used for the real-time PCR. Each reaction contained 5 μl of a 1 in 10 dilution of genomic DNA and was carried out in quadruplicate in a volume of 15 μl in a 384-well LightCycler® 480 PCR plates (Roche), sealed with LightCycler® 480 sealing foil (Roche). Amplification reactions were carried out with Phusion 2X master mix (Thermo Fisher Scientific) using run conditions, primers (0.5 pmol each per reaction) and probe (0.1 pmol per reaction) as described by Furet et al. [55]. Quantitation was done by using standard curves made from known concentrations of linearized plasmid DNA containing the 16S rRNA amplicon. Wells containing nuclease-free water were included as negative controls. Statistical analysis was performed using Graphpad Prism 5, whereby a one-way ANOVA followed by the Tukey test determined statistical significance; ***P value < 0.001, **P value < 0.01, *P value < 0.05.

### Virome DNA extraction and library preparation for MiSeq

DNA corresponding to the viromes of each group of mice was purified from faecal samples, with approximately 1 pellet per mouse included in the extraction. Samples were taken at the timepoints indicated in Additional file 1: Fig. S1. Faecal samples were homogenised in 10 ml SM buffer followed by centrifugation twice at 5000*g* at 10 °C for 10 min and filtration through a 0.45-μm syringe filter to remove particulates and bacterial cells. NaCl (0.5 M final concentration; Sigma) and 10% w/v polyethylene glycol (PEG-8000; Sigma) were added to the resulting filtrate and incubated at 4 °C overnight. Following centrifugation at 5000*g* at 4 °C for 20 min, the pellet was resuspended in 400 μl SM buffer. An equal volume of chloroform (Fisher) was added and following 30 s of vortexing the sample was centrifuged at 2500*g* for 5 min at RT. The aqueous top layer is retained, and it was subjected to RNase I (10 U final concentration; Ambion) and DNase (20 U final concentration; TURBO DNA-free™ Kit, Invitrogen) treatment in accordance with the manufacturer’s guidelines. To isolate DNA, virus like particles were incubated with 20 μL of 10% SDS and 2 μL of proteinase K (Sigma, 20 mg/mL) for 20 min at 56 °C, prior to lysis by the addition of 100 μL of Phage Lysis Buffer (4.5 M guanidine thiocyanate; 45 mM sodium citrate; 250 mM sodium lauroyl sarcosinate; 562.5 mM β-mercaptoethanol; pH 7.0) with incubation at 65 °C for 10 min. Viral DNA was purified by two treatments with an equal volume of phenol:chloroform:isoamyl alcohol (25:24:1) and passing the resulting purified DNA through a QIAGEN Blood and Tissue Purification Kit and eluting samples in 50 μL of AE Buffer. In Study 1, the viral DNA was used directly for Nextera XT library preparation (Illumina) as described by the manufacturer. In Study 2, the DNA concentrations were equalised prior to amplification using an Illustra GenomiPhi V2 kit (GE Healthcare). Amplifications of purified viral DNA were performed in triplicate on all samples as described by the manufacturer. Subsequently, an equal volume of each amplification and an equal volume of the original viral DNA purification were pooled together and used for paired-end Nextera XT library preparation. All samples were sequenced on an Illumina MiSeq at GATC in Germany.

### Analysis of virome sequencing data

The quality of the raw reads was visualised with FastQC v0.11.3. Nextera adapters were removed with cutadapt v1.9.1 [56] followed by read trimming and filtering with Trimmomatic v0.36 [57] to ensure a minimum length of 60, maximum length of 250, and a sliding window that cuts a read once the average quality in a window size of 4 follows below a Phred score of 30. Reads were then assembled with the metaSPAdes assembler [58]. In order for a contig to be included in the final analysis, it must have been at least 5 kb in length, then either detected as viral by Virsorter [59] in the virome decontamination mode or had a significant BLAST hit (95% identity over 95% of the length) to a genome in RefSeq Virus or had no significant BLAST hits (any alignment length with an *e*-value greater than 1e−10) against nt. This allowed us to include known viruses, putative viruses predicted by virsorter and completely novel viral sequences not yet included in any database. The quality-filtered reads were then aligned to this contig set using bowtie2 v2.1.0 [60] using the end to end alignment mode. A count table was generated with samtools v0.1.19 which was then imported into R v3.3.0 where the relative abundance of contigs (labelled as putative viruses) was plotted using ggplot2 v2.2.1.

## Supplementary information

**Additional file 1: Figure S1.** Experimental design of Study 1 (A) and Study 2 (B). BALB/c mice (*n* = 16) were, after acclimatisation, administered antibiotic treatment. After a period of antibiotic wash-out the group was split into two (*n* = 8) and the mice were gavaged with an FVT, a sterile virome faecal filtrate (either viable or heat killed) that had been isolated from frozen faecal samples obtained from the mice during acclimatisation. In Study 2 (B) a second gavage was administered 4 days after the first, dotted vertical lines representing time points within a day. Each group of mice was individually caged. Each solid vertical line represents a day. Time points selected for sampling the faecal microbiota of each mouse in each treatment group are represented as circles and labelled. Samples were subjected to 16S rRNA sequencing and viral metagenomic sequencing.

**Additional file 2: Tables S1- S7. Table S1.** Numbers of MiSeq 16 s *rRNA* amplicon gene sequenced paired reads per sample for Study 1, both pre and post-quality filtering. **Table S2.** Numbers of MiSeq 16 s *rRNA* amplicon gene sequenced paired reads per sample for Study 2, both pre- and post-quality filtering. **Table S3.** Statistical analysis of bacteriome β-diversity of Study 1 and Study 2 Control and FVT groups using Adonis PERMANOVA (Model formula = antibiotic treatment timepoint + Phage/control status). **Table S4.** Statistically significant differentially abundant taxa between Study 1 FVT and Control Mice. OTUs that differ from CT00 mice are highlighted in blue. **Table S5.** Statistically significant differentially abundant taxa between Study 2 FVT and Control Mice. OTUs that differ from CT00 mice are highlighted in blue. **Table S6.** Observed number of contigs/putative viruses in Study 1 and Study 2 mice. **Table S7.** Summary of Study 1 and Study 2 sample IDs, meta data and corresponding accession numbers.

**Additional file 3: Figure S2.** PCoA plots compiled using Bray Curtis and Weighted Unifrac for Study 1 (A) and Study 2 (B). Statistically significant *P* values following UniFrac PERMANOVA analysis performed with the Adonis function to determine the statistical differences between FVT and Control mice have been inserted.

**Additional file 4: Figure S3.** Shannon diversity index was used to display the bacteriome alpha diversity over time for Study 1 (A) and Study 2 (B). No statistical differences were observed in alpha diversity between FVT and Control mice at corresponding time points.

**Additional file 5: Figure S4.** qPCR was used to determine the approximate bacterial cell numbers present per gram of faeces in Study 1 (A) and Study 2 (B). Columns represent samples from individual mice (mean of four technical replicates), coloured to correspond with treatment groups as labelled on the x-axis. Results indicates that bacterial cell numbers dropped dramatically following antibiotic treatment in both studies. A One-Way ANOVA followed by Tukey test determined statistical significance; ***P value< 0.001, ** P value< 0.01, *P value< 0.05.

**Additional file 6: Figure S5.** The abundance of different taxa visualised here at the genus level for Study 1 (A) and Study 2 (B) in mice pre-treatment (Study1: S1-CT000 and S1-CT00; Study 2: S2-CT000 and S2-CT00), post-antibiotic treatment (Study 1: S1-CTAB; Study2: S2-CTAb) and over time post-gavage with either viable bacteriophage (Study 1: S1-V1 to S1-V4; Study 2: S2-V1 to S2-CeV6), or heat treated non-viable bacteriophage (Study 1: S1-C1 to S1-C4; Study 2: S2-C1 to S2-CeC6).

**Additional file 7: Figure S6.** The percentage relative abundance of the differentially abundant OTUs identified (via DESeq2) between FVT and Control mice are displayed to depict their relative contribution to the whole bacterial community in each cohort and in pre-antibiotic treated mice (Study1: S1-CT00 or Study 2: S2-CT00) at the timepoints indicated. OTUs that are found to be statistically different between Control mice and CT00 mice ((S1-CT00 in Study 1 or S2-CT00 Study 2) are highlighted in red text. Those that differ in their abundance to both FVT mice and CT00 mice ((S1-CT00 in Study 1 or S2-CT00 Study 2) and depicted in blue text. It appears that mice that received an FVT are more likely to maintain OTU abundances similar to their pre-treatment state.

## Data Availability

All sequence data used in the analyses were deposited in the Sequence read Archive (SRA) (http://www.ncbi.nlm.nih.gov/sra) under BioProject PRJNA385256 for the 16S rRNA sequence data and BioProject PRJNA385134 for the virome sequence data. Sample IDs, meta data and corresponding accession numbers are summarised in supplementary Additional file 2: Table S7. All raw count tables, 16S taxonomic assignments and R code used for the analysis are available at Figshare repository [61].

## Abbreviations

FVT: Faecal viral transfer
hnFVT: Heat- and nuclease-treated faecal viral transfer
FFT: Faecal filtrate transplant
FMT: Faecal microbial transplantation
PCoA: Principal co-ordinate analysis
IBD: Inflammatory bowel disease
HIV: Human immunodeficiency virus
