# Supplementary figures and images for: Autochthonous faecal viral transfer (FVT) impacts the murine microbiome after antibiotic perturbation
Source: BMC Biol. 2020 Nov 20;18:173. doi: 10.1186/s12915-020-00906-0 (PMC7679995; doi:10.1186/s12915-020-00906-0)

# Study 1

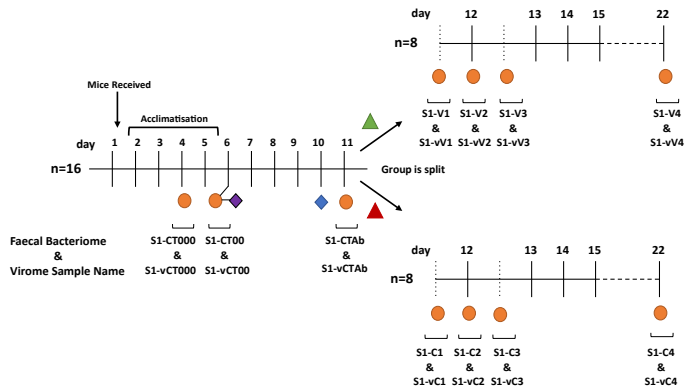

# Study 2

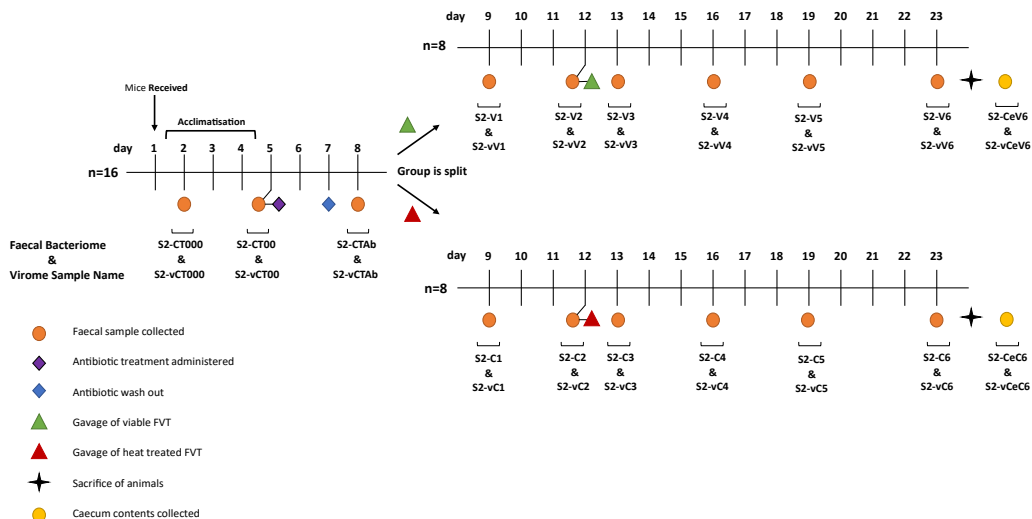

Supplement: Supplementary file 1 — Additional file 1: Figure S1. Experimental design of Study 1 (A) and Study 2 (B). BALB/c mice (n = 16) were, after acclimatisation, administered antibiotic treatment. After a period of antibiotic wash-out the group was split into two (n = 8) and the mice were gavaged with an FVT, a sterile virome faecal filtrate (either viable or heat killed) that had been isolated from frozen faecal samples obtained from the mice during acclimatisation. In Study 2 (B) a second gavage was administered 4 days after the first, dotted vertical lines representing time points within a day. Each group of mice was individually caged. Each solid vertical line represents a day. Time points selected for sampling the faecal microbiota of each mouse in each treatment group are represented as circles and labelled. Samples were subjected to 16S rRNA sequencing and viral metagenomic sequencing. [file 12915_2020_906_MOESM1_ESM.pdf]

**A**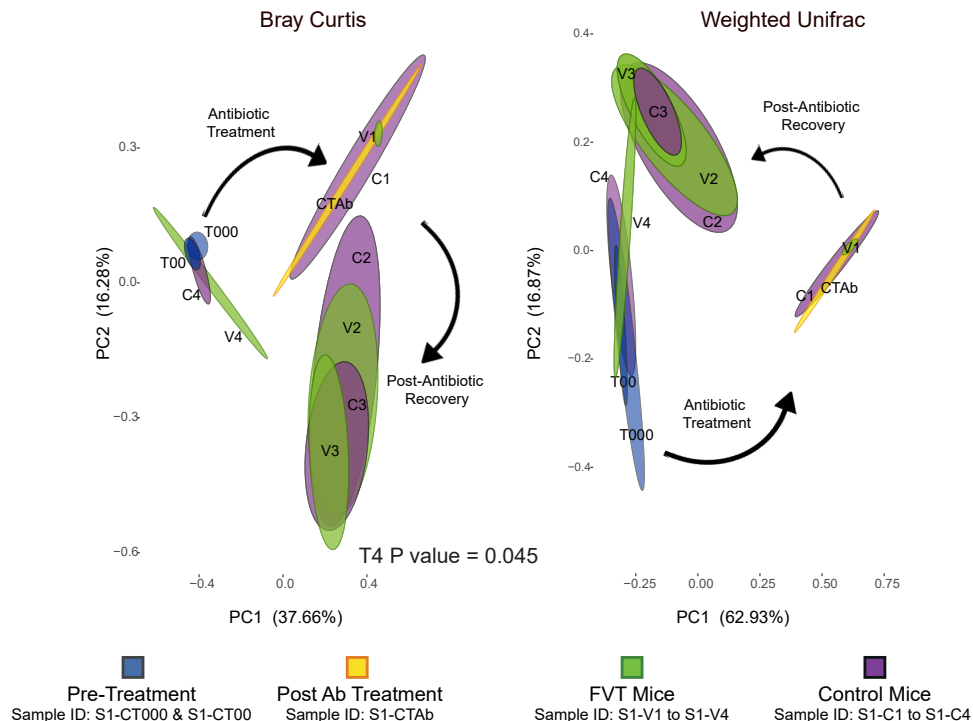**B**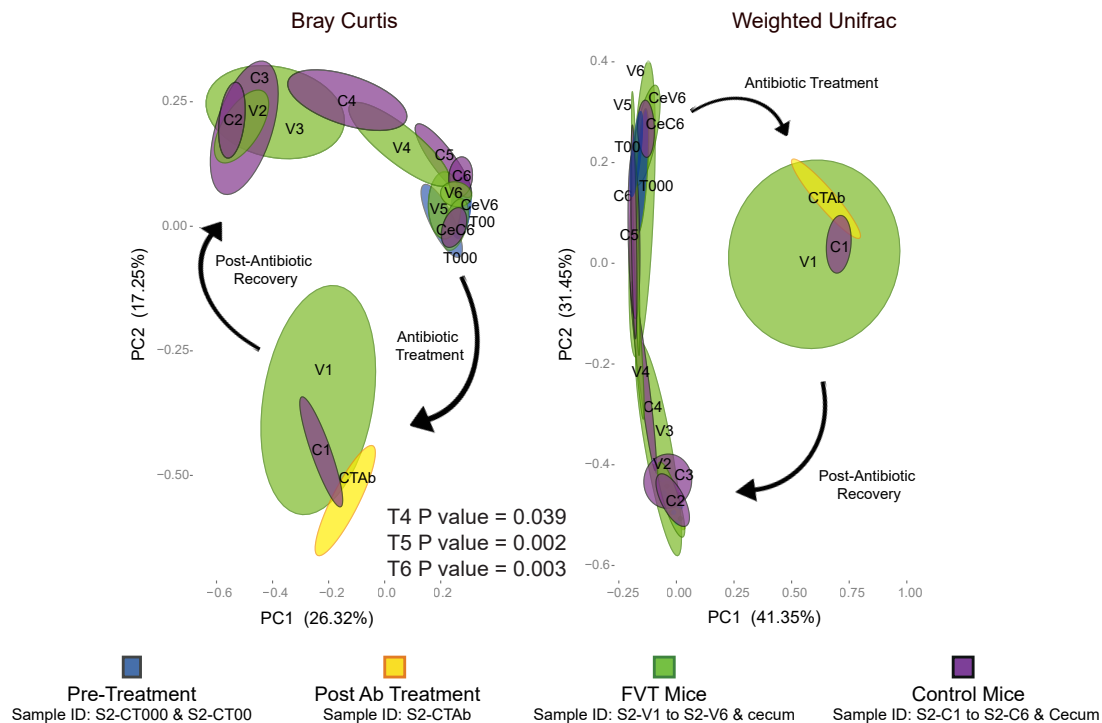

Supplement: Supplementary file 3 — Additional file 3: Figure S2. PCoA plots compiled using Bray Curtis and Weighted Unifrac for Study 1 (A) and Study 2 (B). Statistically significant P values following UniFrac PERMANOVA analysis performed with the Adonis function to determine the statistical differences between FVT and Control mice have been inserted. [file 12915_2020_906_MOESM3_ESM.pdf]

**A**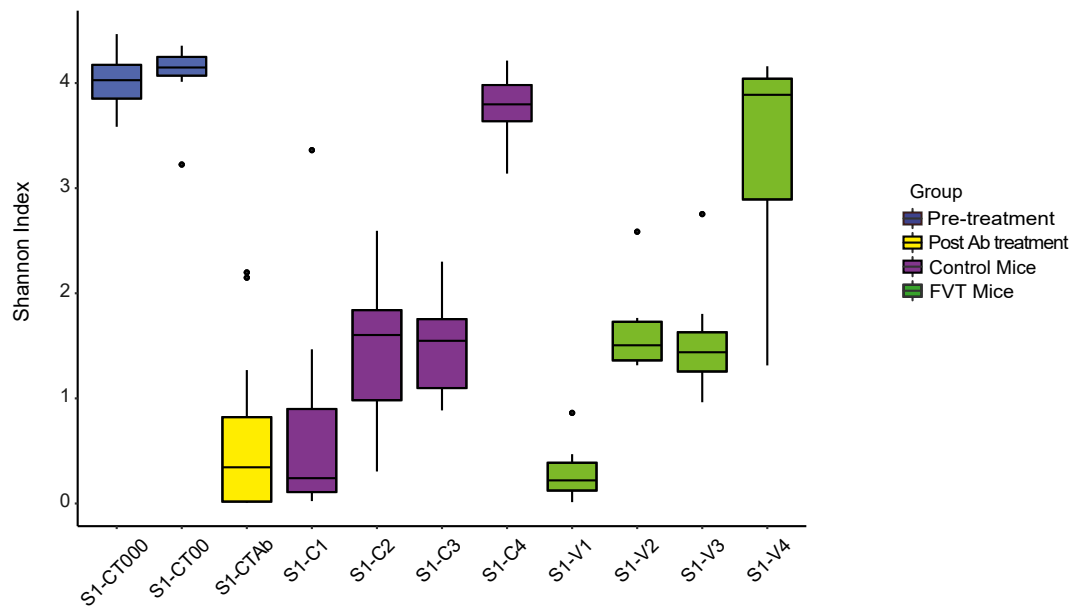**B**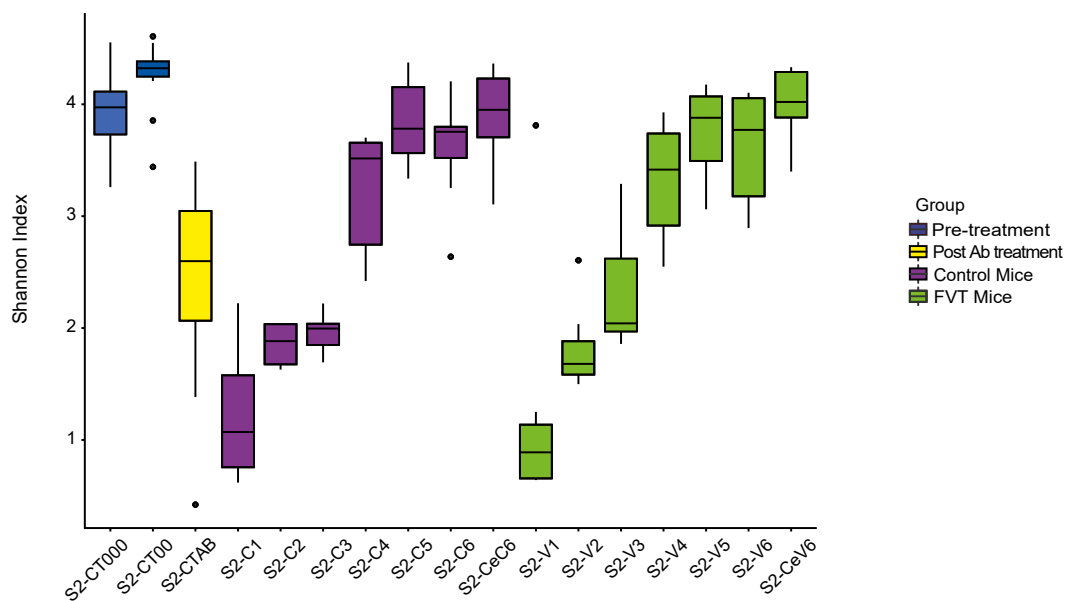

Supplement: Supplementary file 4 — Additional file 4: Figure S3. Shannon diversity index was used to display the bacteriome alpha diversity over time for Study 1 (A) and Study 2 (B). No statistical differences were observed in alpha diversity between FVT and Control mice at corresponding time points. [file 12915_2020_906_MOESM4_ESM.pdf]

**A**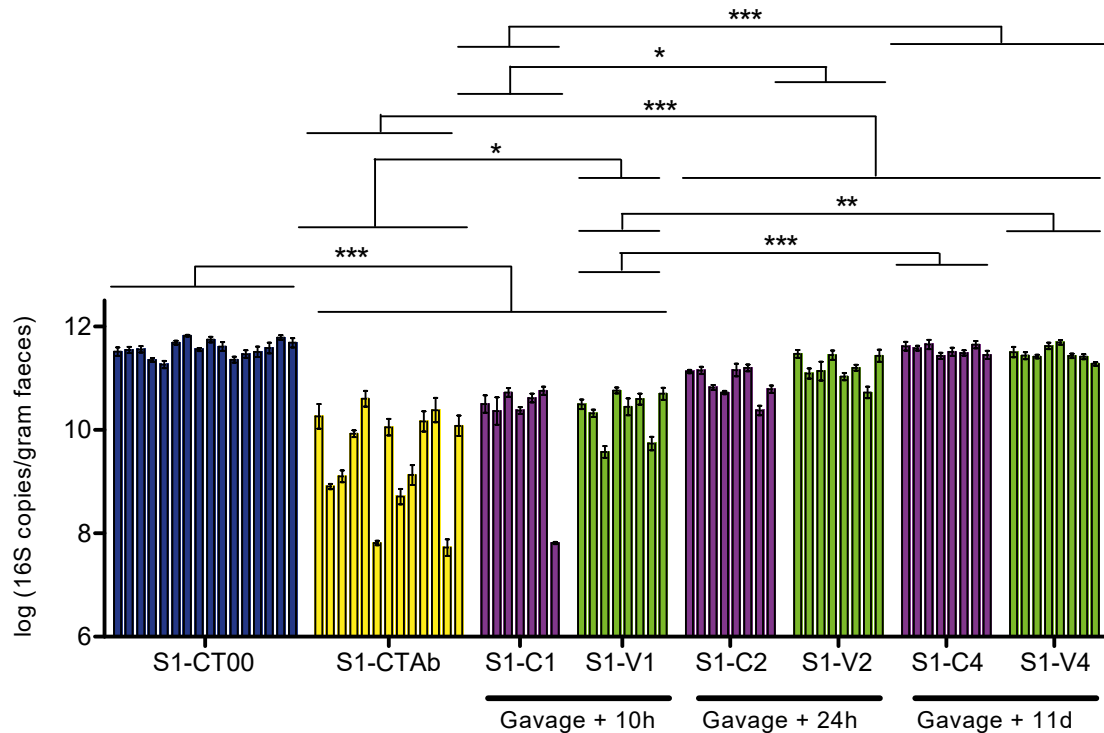**B**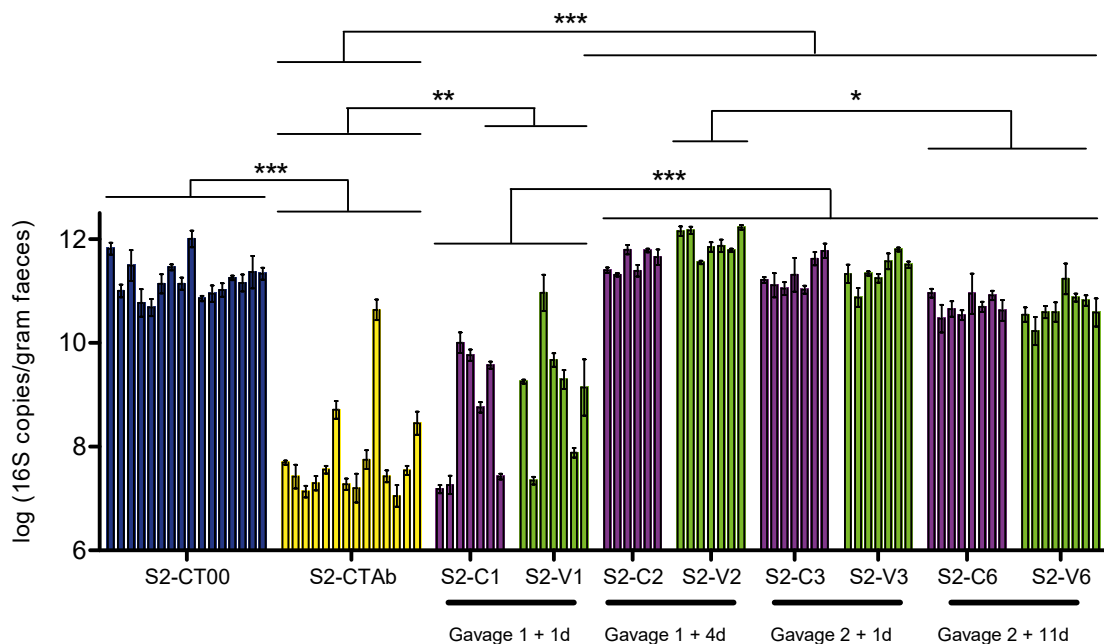

Supplement: Supplementary file 5 — Additional file 5: Figure S4. qPCR was used to determine the approximate bacterial cell numbers present per gram of faeces in Study 1 (A) and Study 2 (B). Columns represent samples from individual mice (mean of four technical replicates), coloured to correspond with treatment groups as labelled on the x-axis. Results indicates that bacterial cell numbers dropped dramatically following antibiotic treatment in both studies. A One-Way ANOVA followed by Tukey test determined statistical significance; ***P value< 0.001, ** P value< 0.01, *P value< 0.05. [file 12915_2020_906_MOESM5_ESM.pdf]

# A Study 1

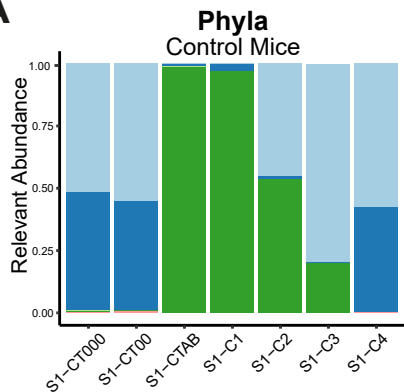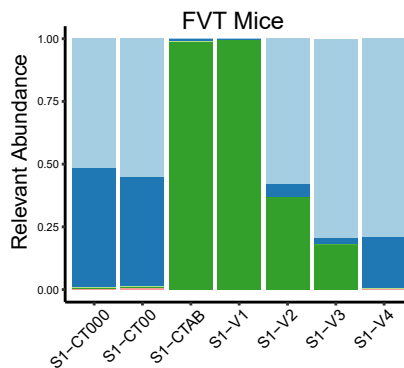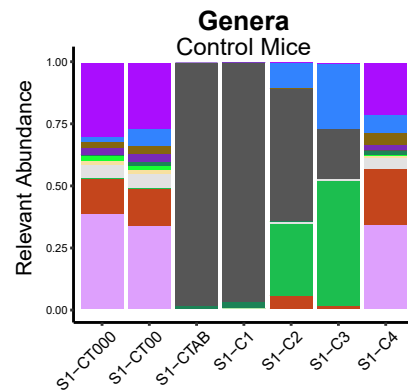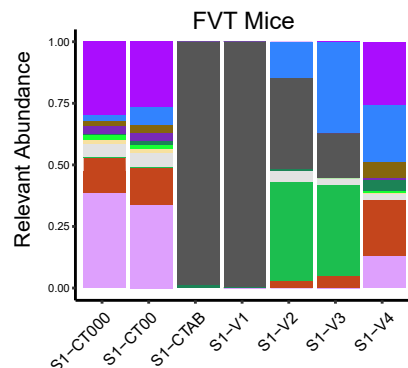

# B Study 2

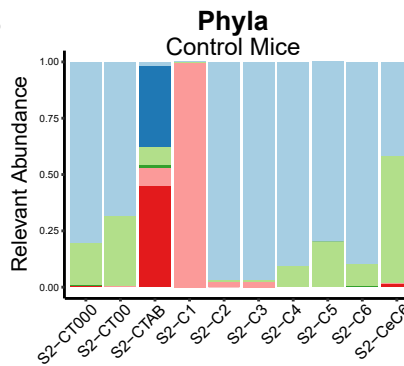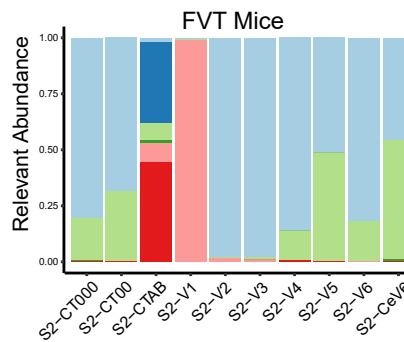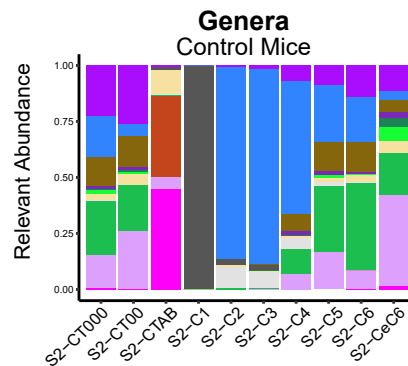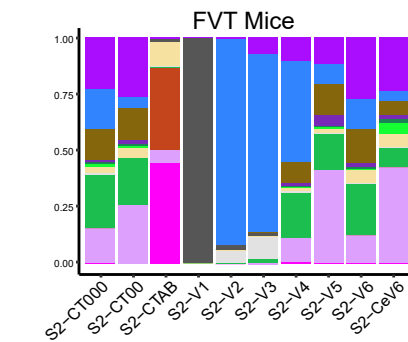

Supplement: Supplementary file 6 — Additional file 6: Figure S5. The abundance of different taxa visualised here at the genus level for Study 1 (A) and Study 2 (B) in mice pre-treatment (Study1: S1-CT000 and S1-CT00; Study 2: S2-CT000 and S2-CT00), post-antibiotic treatment (Study 1: S1-CTAB; Study2: S2-CTAb) and over time post-gavage with either viable bacteriophage (Study 1: S1-V1 to S1-V4; Study 2: S2-V1 to S2-CeV6), or heat treated non-viable bacteriophage (Study 1: S1-C1 to S1-C4; Study 2: S2-C1 to S2-CeC6). [file 12915_2020_906_MOESM6_ESM.pdf]
