# Supplementary material for: Autochthonous faecal viral transfer (FVT) impacts the murine microbiome after antibiotic perturbation
Source: BMC Biol. 2020 Nov 20;18:173. doi: 10.1186/s12915-020-00906-0 (PMC7679995; doi:10.1186/s12915-020-00906-0)
Supplement: Supplementary file 7 — Additional file 7: Figure S6. The percentage relative abundance of the differentially abundant OTUs identified (via DESeq2) between FVT and Control mice are displayed to depict their relative contribution to the whole bacterial community in each cohort and in pre-antibiotic treated mice (Study1: S1-CT00 or Study 2: S2-CT00) at the timepoints indicated. OTUs that are found to be statistically different between Control mice and CT00 mice ((S1-CT00 in Study 1 or S2-CT00 Study 2) are highlighted in red text. Those that differ in their abundance to both FVT mice and CT00 mice ((S1-CT00 in Study 1 or S2-CT00 Study 2) and depicted in blue text. It appears that mice that received an FVT are more likely to maintain OTU abundances similar to their pre-treatment state. [file 12915_2020_906_MOESM7_ESM.pdf]

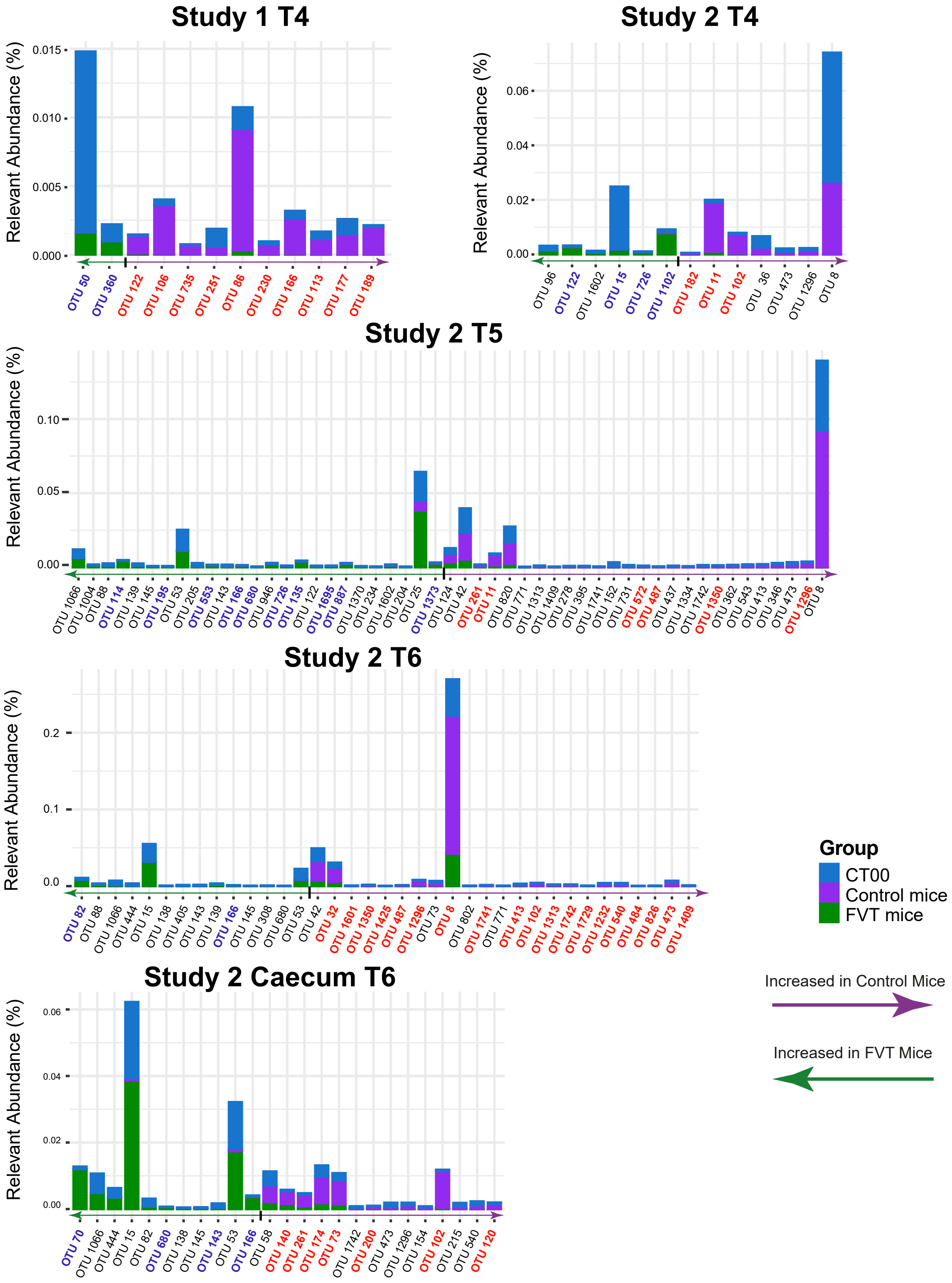

Study 1 T4
Relevant Abundance (%)
0.000
0.005
0.010
0.015
OTU 50
OTU 360
OTU 122
OTU 106
OTU 735
OTU 251
OTU 86
OTU 230
OTU 166
OTU 113
OTU 177
OTU 189
Study 2 T4
Relevant Abundance (%)
0.00
0.02
0.04
0.06
OTU 96
OTU 122
OTU 1602
OTU 15
OTU 726
OTU 1102
OTU 182
OTU 11
OTU 102
OTU 36
OTU 473
OTU 1296
OTU 8
Study 2 T5
Relevant Abundance (%)
0.00
0.05
0.10
OTU 1066
OTU 1004
OTU 88
OTU 114
OTU 139
OTU 145
OTU 195
OTU 53
OTU 205
OTU 553
OTU 143
OTU 166
OTU 680
OTU 946
OTU 726
OTU 135
OTU 122
OTU 1695
OTU 887
OTU 1370
OTU 234
OTU 1602
OTU 204
OTU 25
OTU 1373
OTU 124
OTU 42
OTU 261
OTU 11
OTU 820
OTU 771
OTU 1313
OTU 1409
OTU 278
OTU 395
OTU 1741
OTU 152
OTU 731
OTU 572
OTU 487
OTU 437
OTU 1334
OTU 1742
OTU 1350
OTU 362
OTU 543
OTU 413
OTU 346
OTU 473
OTU 1296
OTU 8
Study 2 T6
Relevant Abundance (%)
0.0
0.1
0.2
OTU 82
OTU 88
OTU 1066
OTU 444
OTU 15
OTU 138
OTU 405
OTU 143
OTU 139
OTU 166
OTU 145
OTU 308
OTU 680
OTU 53
OTU 42
OTU 32
OTU 1601
OTU 1350
OTU 1425
OTU 487
OTU 1296
OTU 73
OTU 8
OTU 802
OTU 1741
OTU 771
OTU 413
OTU 102
OTU 1313
OTU 1742
OTU 1729
OTU 1232
OTU 540
OTU 484
OTU 826
OTU 473
OTU 1409
Group
CT00
Control mice
FVT mice
Study 2 Caecum T6
Relevant Abundance (%)
0.00
0.02
0.04
0.06
OTU 70
OTU 1066
OTU 444
OTU 15
OTU 82
OTU 680
OTU 138
OTU 145
OTU 143
OTU 53
OTU 166
OTU 58
OTU 140
OTU 261
OTU 174
OTU 73
OTU 1742
OTU 200
OTU 473
OTU 1296
OTU 154
OTU 102
OTU 215
OTU 540
OTU 120
Increased in Control Mice
Increased in FVT Mice
